# Supplementary figures and images for: Species-specific circular RNA circDS-1 enhances adaptive evolution in Talaromyces marneffei through regulation of dimorphic transition
Source: PLoS Genet. 2025 Mar 6;21(3):e1011482. doi: 10.1371/journal.pgen.1011482 (PMC11928065; doi:10.1371/journal.pgen.1011482)

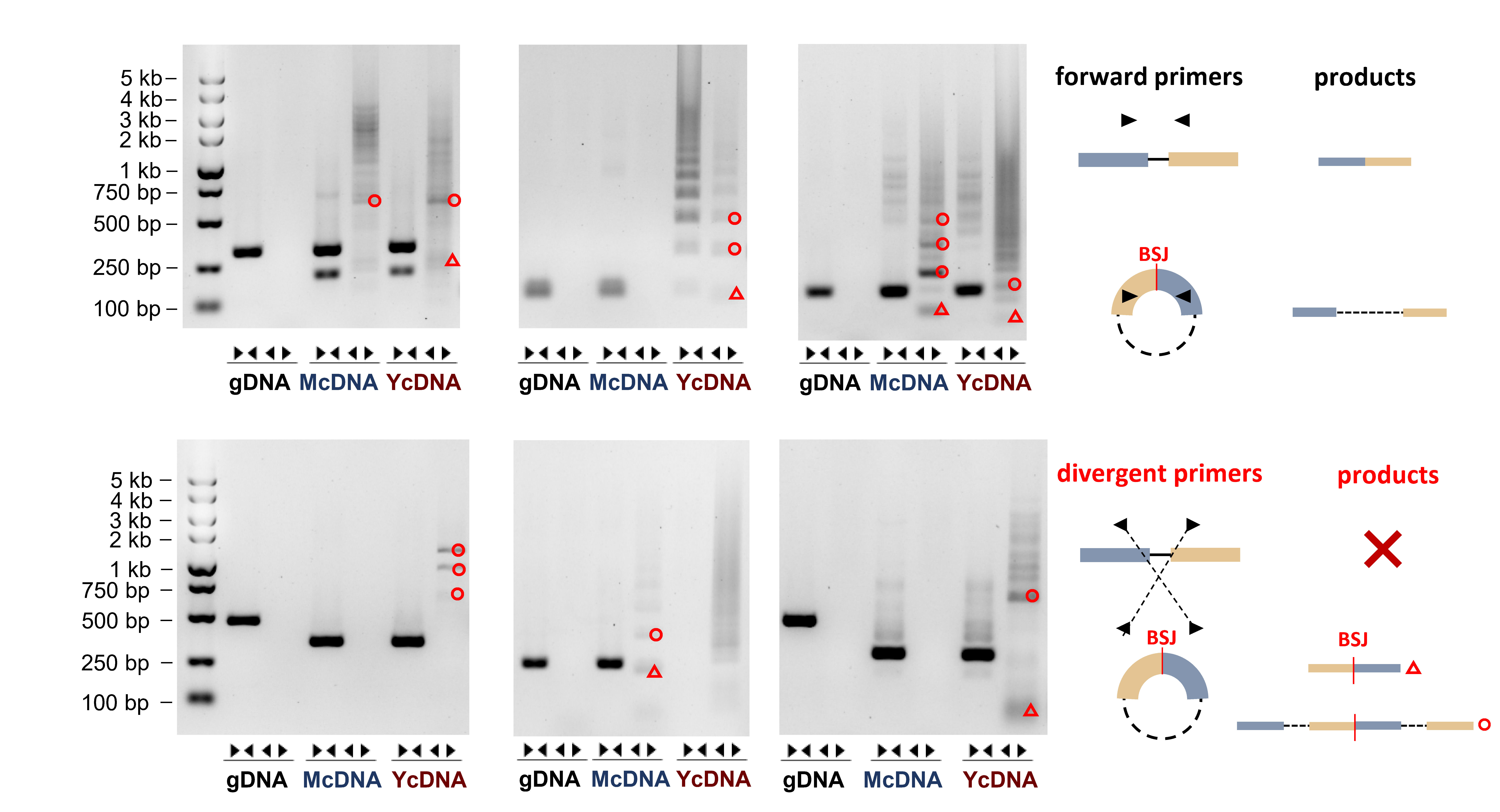

Supplement: S1 Fig — Divergent primers could amplify BSJ of circRNAs, thus products could be detected in mycelia and yeast cDNA (McDNA and YcDNA) but not genomic DNA (gDNA). Forward primers were designed as controls to exclude trans rearrangement events at the genomic level that may mimic BSJ sequences. BSJ is represented by a red triangle, and the red circle represents the amplification product of “BSJ+full length*n”, which is different from linear RNA. (TIF) [file pgen.1011482.s001.tif]

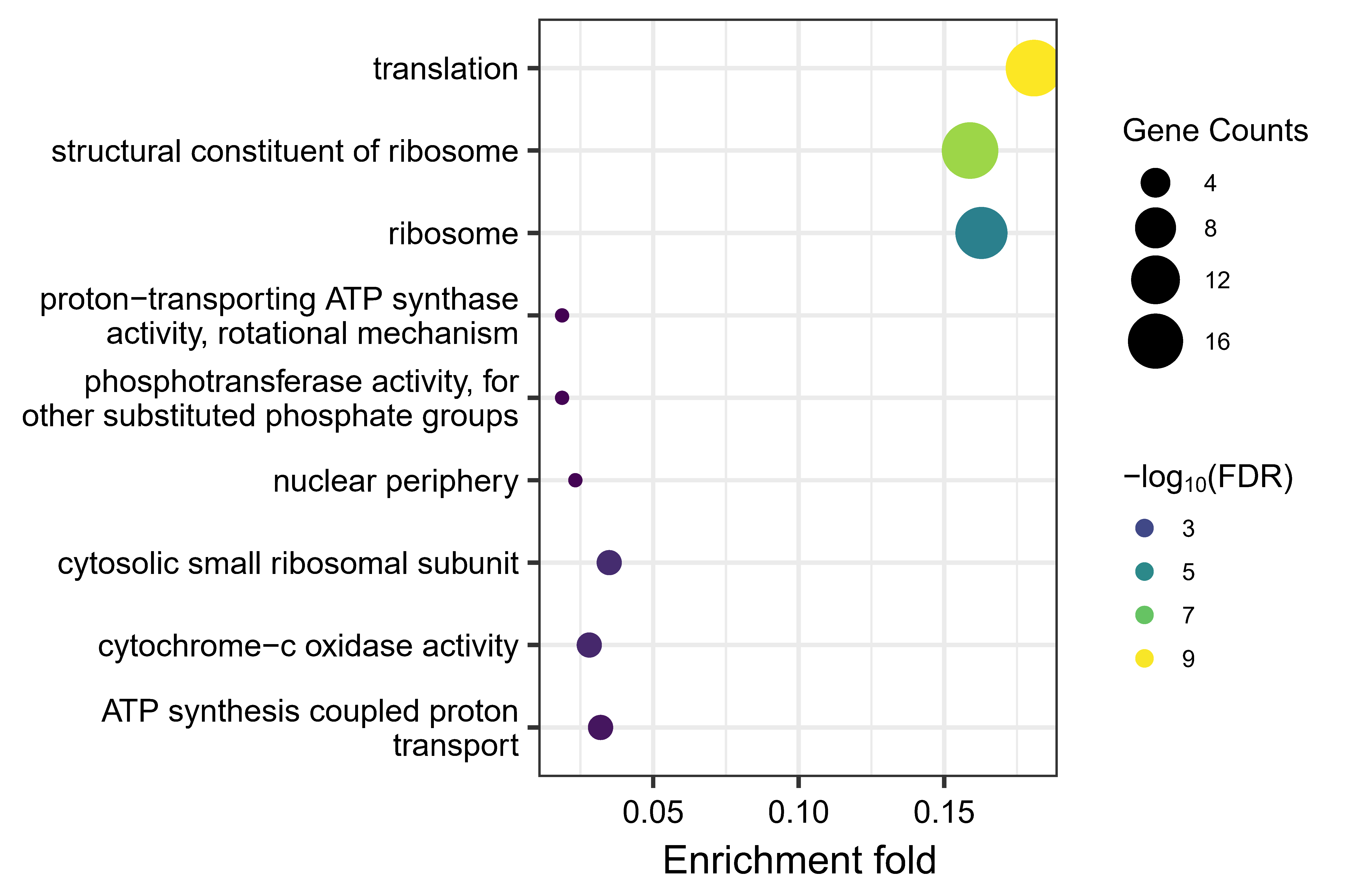

Supplement: S2 Fig — The y-axis is the enriched GO terms. The size of the dot represents the identified gene counts. (TIF) [file pgen.1011482.s002.tif]

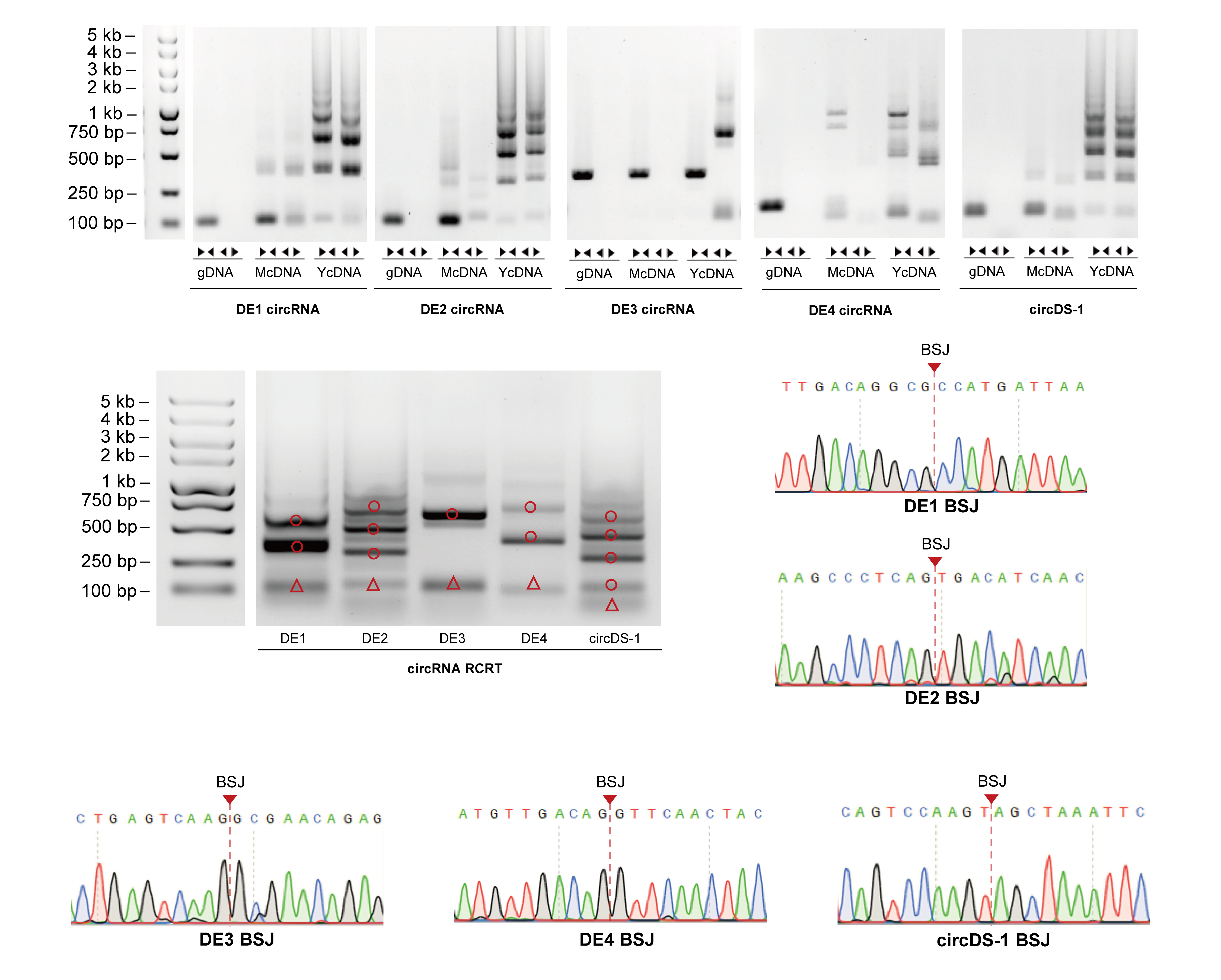

Supplement: S3 Fig — The products of BSJs are indicated by red triangles, while the red circles represent the amplified products that occur specifically in circRNAs but not in linear RNAs. (TIF) [file pgen.1011482.s003.tif]

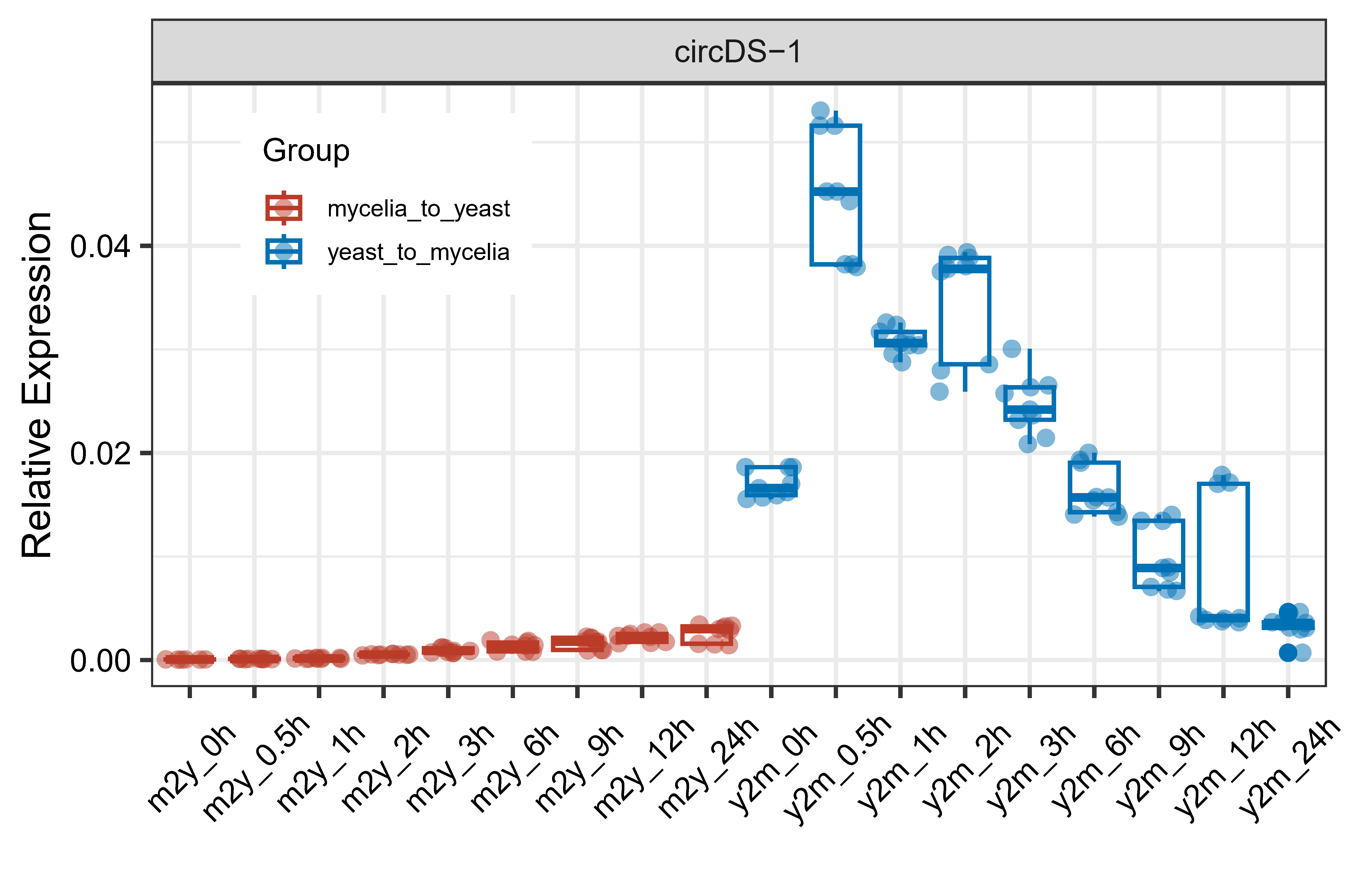

Supplement: S4 Fig — Three biological replicates and three technical replicates for each time point, and the relative expression was calculated using the 2-ΔΔCt method, with actin as the internal reference gene. (TIF) [file pgen.1011482.s004.tif]

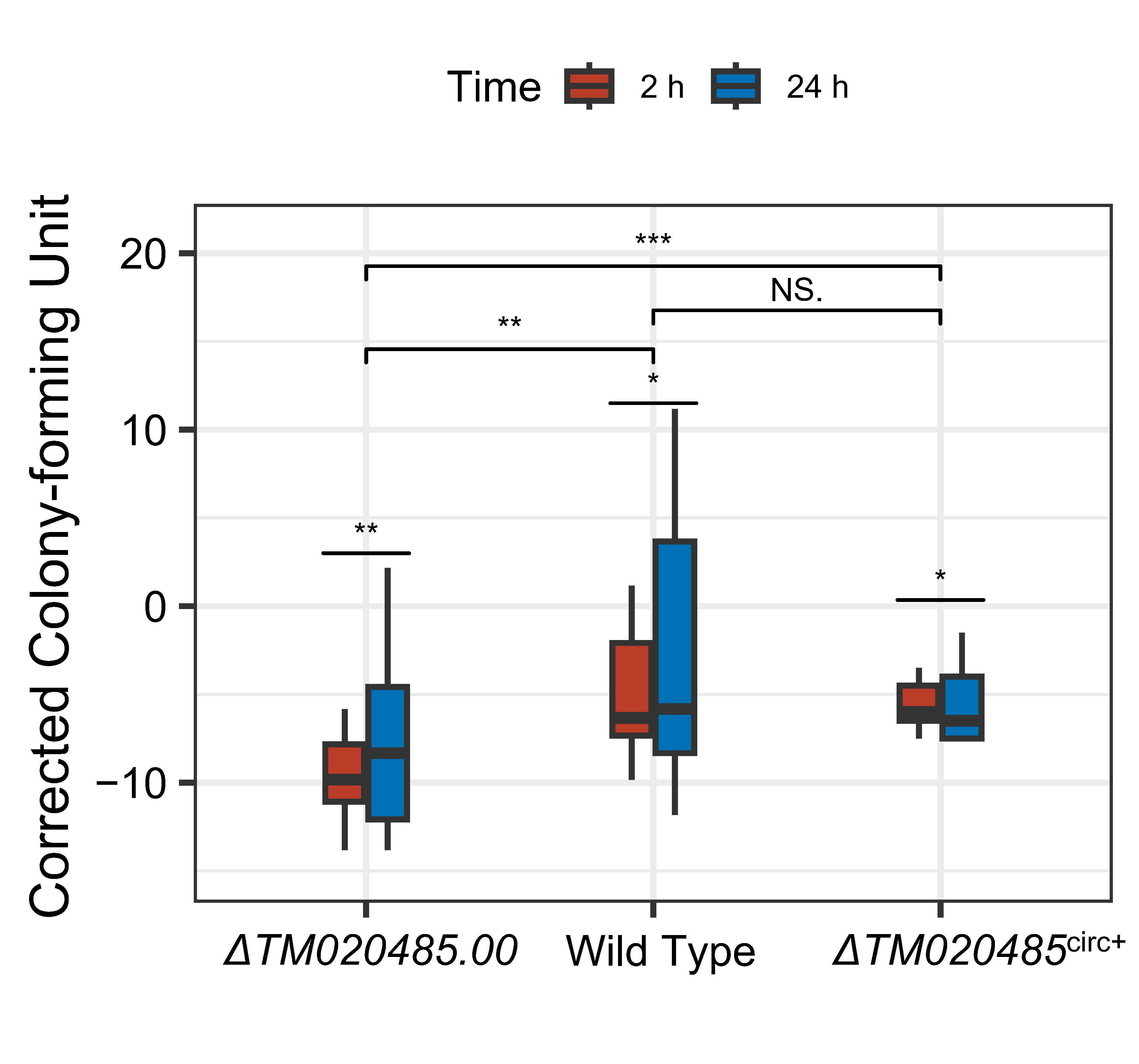

Supplement: S5 Fig — (TIF) [file pgen.1011482.s005.tif]

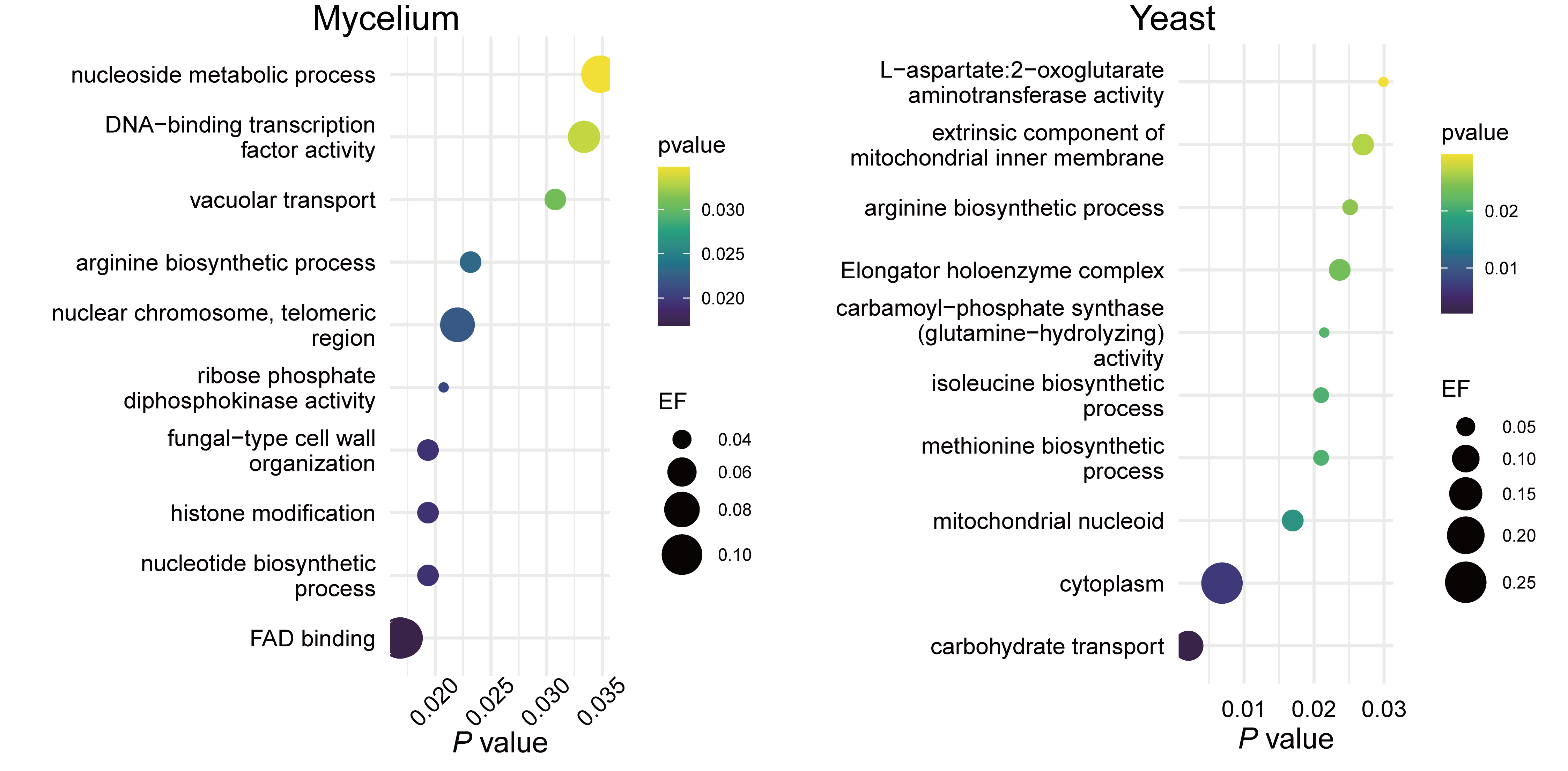

Supplement: S6 Fig — The y-axis is the enriched GO terms. The size of the dot represents the identified enrichment fold. (TIF) [file pgen.1011482.s006.tif]

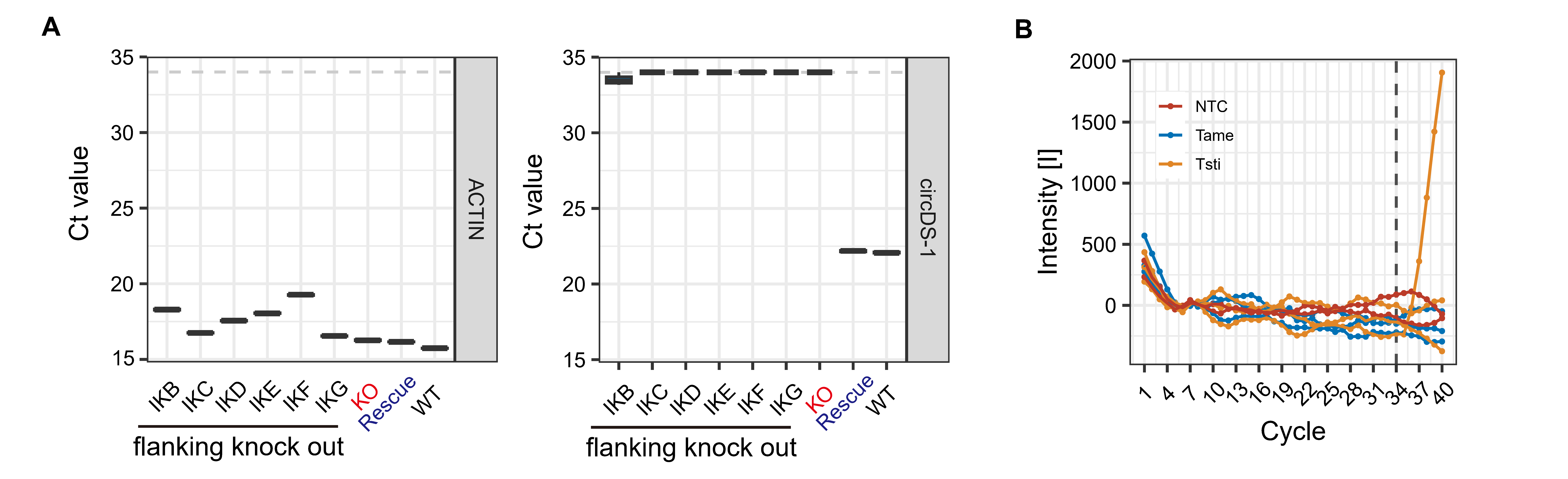

Supplement: S7 Fig — A, Ct values of reference gene actin and circDS-1 in specific region knockouts (IK), ΔTM020485, ΔTM020485 rescued with circDS-1 (ΔTM020485circ+) and wild type strains. B, Amplification signals of circDS-1 in T. stipitatus and T. amestolkiae. (TIF) [file pgen.1011482.s007.tif]
