# Supplementary material for: Species-specific circular RNA circDS-1 enhances adaptive evolution in Talaromyces marneffei through regulation of dimorphic transition
Source: PLoS Genet. 2025 Mar 6;21(3):e1011482. doi: 10.1371/journal.pgen.1011482 (PMC11928065; doi:10.1371/journal.pgen.1011482)
Supplement: Attachment — Submitted filename: point-by-point response.docx [file pgen.1011482.s011.docx]

We sincerely appreciate the reviewers’ insightful comments and constructive suggestions, which have helped us significantly improve the quality of our manuscript. Below, we provide point-by-point responses to address all concerns raised, and all the changes are highlighted in the revised manuscript.

**Reviewer #1**

**Using a new sequencing strategy (NreT-seq), the authors investigate the conservation of circRNAs in *T. marneffei*. Through differential expression analysis, they find that circRNAs are involved in the regulation of the dimorphic transition of *T. marneffei*, with circDS-1 exhibiting the highest fold change in differential transcription. They further discover that circDS-1 is less expressed in mycelia and more expressed in yeast, and that down-regulation of circDS-1 inhibits the mycelia-to-yeast transition. Moreover, the *T. marneffei*-specific flanking sequence contributes to the generation of circDS-1, which may utilize an “intron definition” splicing model. Overall, the data are of high quality and judiciously interpreted. I have only a few minor comments that might help further clarify this important finding.**

**Specific comments:**

**Q1. As shown in Figure 4B, the expression level of circDS-1 in OE#rep1 was higher than in OE#rep2 and rep3 (labeled rep2), and the morphogenesis of OE#rep1 was noticeably different from that of OE#rep2 and rep3. The morphology of OE#rep1 resembled mycelia more closely. Does further overexpression of circDS-1 lead to changes in mycelial morphology?**

Sorry for the misalignment of the label of replicate name and we have revised Figure 4B. We appreciate you for asking this insightful question. In fact, in our overexpression strain construction, we obtained more than 6 strains with circDS-1 overexpression. Probably due to the complex circularization regulation mechanism, the expression levels of these overexpression strains have certain differences. In order to show the interference of abnormal expression of circDS-1 on physiological effects, we selected the three strains with the highest expression levels for display in our results (rep#1, rep#2 and rep#3), while the slightly lower-level overexpression strains that were not shown in the manuscript showed slight phenotypes (rep#4, rep#5 and rep#6 in the figure below). The expression level and phenotype of rep#4 were like those of rep#3, while rep#5 and rep#6, which had lower expression, only showed abnormal burr-like morphology at the edge of the colony, and the microscopic morphology of these burrs was like that of rep#2 and rep#3. This indeed shows that the overexpression effect of circDS-1 is dose-dependent to a certain extent, and a certain threshold must be reached to cause obvious phenotypic changes in yeast condition.

Among these overexpression strains, rep#1 is the strain with the highest expression level. This expression level may be limited by the efficiency of our expression vector and the permissive threshold of the regulatory network of circDS-1. Therefore, although we tried more overexpressed strains, we failed to obtain transformants with a higher level than rep#1. Higher levels of circDS-1 may require further optimization of the circRNA overexpression system for fungal splicing regulation, which is also our next research direction. We will also discuss this phenomenon in the discussion section (line 411-416).

**Q2. The relative quantification (RQ) of circDS-1 in mycelia and yeast is presented. In my view, the authors should test the differential expression of circDS-1 during the transition from mycelia to yeast or from yeast to mycelia.**

Thank you for your valuable suggestions. Since the time series data used in Figure 7C of the manuscript are from the poly(A) tail selection library, they cannot be used to detect the dynamic expression of circDS-1 during the dimorphic transition. Therefore, we performed RT-qPCR quantification of circDS-1, *TM11485.00* and *TM050570.00* (two KH domain genes) in wild-type liquid culture, and the process was the same as Figure 5 (Method: Dynamic dimorphic transition section). Since circDS-1 has a regulatory effect on yeast retarded transformation, and the molecular effect generally precedes the appearance of the phenotypic effect, we focused the detection on the early time points of the dimorphic transition, i.e., 0, 0.5, 1, 2, 3, 6, 9, 12, 24 h, to capture the expression changes of circDS-1 at this stage. The results showed that circDS-1 showed a steady upward trend in the early stage of the transformation from mycelium to yeast, and a rapid upregulation and then a significant decline in the early stage of the transformation from yeast to mycelium. This molecular change ahead of the phenotype indeed confirms the regulatory role of circDS-1 in the dimorphic transformation. Moreover, this trend in the quantitative results is the same as that of *TM11485.00* and *TM050570.00*, which also confirms our hypothesis in figure 7C that the generation of circDS-1 is related to *TM11485.00* and *TM050570.00*.

It should also be noted that the above quantitative results are from the time points of liquid culture, while the time series data used in Figure 7C of the previous manuscript are the results of solid culture. Therefore, there are differences in the time point and absolute expression values between the two results, but the overall trend is consistent. In order to avoid confusion, we replaced the time series in Figure 7C in the original text with the latest RT-qPCR results. The time expression results of circDS-1 are also provided as Supplementary Figure 4. We also added an explanation of this new result in the manuscript (line 253-259).

**Q3. In Figure 4, the authors should clarify the meaning of the purple arrow.**

We apologize for not being able to make the figure legend clearer and we have supplemented it in the manuscript (line 968-969).

**Q4. The authors should illustrate the dynamic dimorphic transition from mycelia to yeast and the mycelia of the wild type of circDS-1^OE^.**

Thank you for your valuable suggestions. We performed dynamic transformation of circDS-1 overexpression strains according to the same method as in the article (Method: Dynamic dimorphic transition section) and found that circDS-1 overexpression blocked hyphal development in the hyphal phase and promoted yeast transformation in the yeast phase. This result is consistent with the results of static circDS-1 overexpression, indicating that high levels of circDS-1 do have a role in promoting yeast cells. This experimental result was added in Figure 5. We also added the explanation of this result in the manuscript (line 266-275).

**Q5. The resolution of the image in Figure 5 is low.**

We are sorry that there is a problem with the image we uploaded. We have updated it with a higher resolution image in the revised version.

**Q6. The authors stated “We then deleted the** **species-specific region while retaining the remaining flanking sequences, which resulted in the complete inhibition of circDS-1 circularization in *T. marneffei*.” The sequence should be illustrated, and the results should be provided, as they are important evidence to support the conclusion.**

We apologize for the inappropriate descriptions in the manuscript. We only used colors to mark the species-specific flanking sequence and KH domain binding sites in the figure and legend (yellow and blue in Figure 7A), but did not provide appropriate references in the context. Therefore, we supplemented the knocked-out sequence information (line 305/577-578) in the text and in the method “Construction of insertion sequences deletion vector”, and added the knockout quantitative results of the highly mutated sequence as Supplementary Figure 7. We obtained a total of 6 strains with successful knockout of highly mutated sequences (IK), and the quantitative results showed that sequence knockout in these 6 strains could completely inhibit the production of circDS-1 (A). In addition, we also supplemented the quantitative results of circDS-1 in closely related species (*Talaromyces amestolkiae* and *Talaromyces stipitatus*) that lack the hypermutated flanking sequence (B). These two strains have no quantitative signals of circDS-1, indicating that neither of them produces circDS-1.

**Reviewer #2:**

**Q1. The data of circDS-1 in TM-infected mouse model is expected.**

We sincerely appreciate your insightful and professional suggestion. Indeed, a mouse virulence experiment would further confirm the regulatory role of circDS-1 on the virulence of *T. marneffei*, and virulence assays would offer stronger evidence than macrophage co-culture experiments. However, our research focuses mainly on the regulation of dimorphic transition by species-specific circDS-1 in *T. marneffei*, with a special emphasis on whether this novel noncoding RNA plays a role in the morphogenesis of *T. marneffei*. To investigate this, we employed a range of morphological experiments to verify the regulatory effect of circDS-1 on both static and dynamic transitions. Our findings indicate that circDS-1 inhibits hyphal formation, maintains the yeast form, and promotes the production of arthrospores, thus confirming its critical role in regulating the dimorphic transition.

Given that the dimorphic transition of *T. marneffei* is closely associated with its virulence, we conducted exploratory macrophage co-culture experiment to explore the relationship between circDS-1 and virulence. While our preliminary observations revealed modulatory effects of circDS-1 on pathogenic traits, it should be emphasized that the elucidation of virulence mechanisms falls beyond the principal objectives of this investigation, which specifically focuses on decoding circDS-1's governance in fungal dimorphism. Notably, the mechanistic basis underlying circDS-1-mediated macrophage survival enhancement remains an open question meriting dedicated future exploration. Although murine models could theoretically reinforce correlative evidence between circDS-1 and virulence phenotypes, such an approach would not substantively advance our core mission to delineate the molecular circuitry of circDS-1 in dimorphic regulation. Therefore, guided by the ethical imperatives of the 3Rs framework (Replacement, Reduction, Refinement) that shape responsible experimental design, we did not conduct the mouse virulence model in our current work. Given the strength of the virulence evidence at this time, we have removed the word "virulence" from the title of the article and changed it to " Species-specific circular RNA circDS-1 enhances adaptive evolution in *Talaromyces marneffei* through regulation of dimorphic transition".

Furthermore, our future work will focus on elucidating the mechanisms through which circDS-1 regulates the dimorphic transition. We believe that once the mechanism is more thoroughly clarified, a comprehensive mouse virulence study will be essential to confirm the regulatory role of circDS-1 and further illuminate its significance in the adaptation and virulence of *T. marneffei*.

**Reviewer #3:**

**Q1. This study is robust and valuable, providing new evidence regarding circRNA. However, the evaluation of genotype and phenotype characteristics appears to be insufficient. Would it be feasible to assess pathogenicity using simple animal models, such as** ***Galleria mellonella* larvae? This approach could further enhance the significance of the research.**

Thank you for your valuable suggestion. We agree that utilizing animal models, such as *Galleria mellonella* larvae, to assess pathogenicity could provide important supplementary data. In fact, we initially conducted experiments using *Galleria mellonella* larvae based on established protocols^1^ to investigate the potential function of circDS-1 in virulence. However, we encountered several challenges with this model, including variability in larval health and inherent biological inconsistencies, which resulted in preliminary data that did not show significant differences between groups.

Specifically, in our experiments, larvae infected with yeast-phase cells (incubated at 37°C) did not exhibit mortality (one larva even progressed to the pupal stage). In contrast, when infected with spores (incubated at 25°C), only two larvae from the deletion strain group died (out of 16 per group), while the majority survived. Additionally, we observed that silk spinning commenced significantly earlier at 25°C compared to 37°C, which introduced confounding factors and complicated the interpretation of the results.

Despite these challenges, we maintain that the role of circDS-1 in regulating the dimorphic transition remains robust, and the core conclusions of our study are unaffected. To address these limitations, we have revised the title of the manuscript to reflect our findings, removing the term “virulence” and emphasizing the role of circDS-1 more accurately in dimorphic transition and adaptive evolution: “Species-specific circular RNA circDS-1 enhances adaptive evolution in *Talaromyces marneffei* through regulation of dimorphic transition”. In future work, we plan to further explore the regulatory mechanisms linking circDS-1 to virulence, potentially employing more stable or alternative models to validate these findings. We appreciate your insightful suggestion and believe that these additional investigations will further strengthen the significance of our research.

**Q2. Another point of consideration is the morphological transformation of *Talaromyces marneffei*, particularly in the yeast phase, which seems to remain unclear and difficult to distinguish from its morphological characteristics. The development of a tool to confirm yeast phase specific markers is crucial. If such confirmation cannot be performed in the laboratory, it would be important to discuss this limitation.**

We fully agree with your comments. Indeed, the development of reliable morphological markers for *T. marneffei* has lagged that of other well-studied fungi, such as *Candida albicans*. In our study, we initially explored the classic yeast-specific *mp1* gene^2^ (*TM070120.00*). While empirical validation confirmed *mp1* utility as a static marker of yeast, our quantitative analyses revealed its inherent limitations in resolving dynamic states (figure below).

As a result, we relied primarily on morphological characteristics as a more consistent and dependable basis for morphic determination in this study. For future work, we plan to leverage whole transcriptome data to identify and validate marker genes specific to the hyphal and yeast conditions, which could potentially serve as fluorescent probes for more precise stage identification. We have now addressed this limitation and outlined our future directions in the Discussion section (line 416-424).

**Q3. Furthermore, the current understanding of the pathogenicity of *Talaromyces marneffei* has shifted. Additional discussion on the Damage Response Framework (DRF) is necessary, as the findings in this study provide significant support for this concept.**

Thank you for acknowledging the significance of the current study. We fully agree with the importance of the DRF in understanding the pathogenicity of *T. marneffei* based on non-coding RNA field. We will include a discussion of the DRF in the revised manuscript and further explain how our findings provide support for this framework (line 380-396).

**Reference**

1. Huang X, Li D, Xi L, Mylonakis E. *Galleria mellonella* Larvae as an Infection Model for *Penicillium marneffei*. ***Mycopathologia***. 2015 Oct;180(3-4):159-64.
2. Woo PC, Lau SK, Lau CC, Tung ET, Chong KT, Yang F, Zhang H, Lo RK, Cai JP, Au-Yeung RK, Ng WF, Tse H, Wong SS, Xu S, Lam WH, Tse MK, Sze KH, Kao RY, Reiner NE, Hao Q, Yuen KY. Mp1p Is a Virulence Factor in *Talaromyces (Penicillium) marneffei*. ***PLoS Negl Trop Dis***. 2016 Aug 25;10(8):e0004907.
